# Interactive effects of age and inflammation on change in ecologically-assessed cognitive functioning

**DOI:** 10.1093/geroni/igab046.2626

**Published:** 2021-12-17

**Authors:** Christopher Engeland, Erik Knight, Martin Sliwinski, Jennifer Graham-Engeland

**Affiliations:** 1 Penn State University, University Park, Pennsylvania, United States; 2 University of Colorado Boulder, Boulder, Colorado, United States; 3 The Pennsylvania State University, University Park, Pennsylvania, United States

## Abstract

Inflammation has been implicated as a precursor to steeper declines in age-associated cognitive decline. Here we investigated biomarkers of peripheral inflammation [basal cytokines, stimulated cytokines (ex vivo), C-reactive protein (CRP)] as moderators of age-related changes in cognitive functioning. As part of the Effects of Stress on Cognitive Aging, Physiology, and Emotion (ESCAPE) study, participants (N = 233; 65% female; 63% Black, 25% Hispanic; 25-65 years of age) completed up to four instances of ambulatory cognitive testing per day across two weeks, over three waves of annual assessments. After each 2-week ecological momentary assessment (EMA) burst, blood was collected and assayed for inflammatory biomarkers. Performance on spatial working memory (mean Euclidean distance errors), processing speed (mean symbol search reaction time), and working memory (n-back test accuracy) tasks were averaged across all instances within an EMA burst. CRP and age interactively predicted change in spatial working memory (B = 0.003, [0.000, 0.005], t(133.60) = 2.350, p = 0.020) such that higher CRP at older ages (~60 years) was associated with a loss of the expected practice effects across waves; at younger ages, CRP did not relate to change in spatial working memory. In a similar fashion, basal (B = -0.002, [-0.004, -0.000], t(103.26) = -2.399, p = 0.018) and stimulated cytokine levels (B = -0.002, [-0.004, -0.000], t(126.65) = -2.183, p = 0.031) interacted with age to predict change in processing speed across waves. These results indicate that inflammation may be critically associated with changes in cognitive functioning in older mid-life adults.

